# Genomic Analyses of Methicillin-Resistant *Staphylococcus pseudintermedius* from Companion Animals Reveal Changing Clonal Populations, Multidrug Resistance, and Virulence

**DOI:** 10.3390/antibiotics13100962

**Published:** 2024-10-12

**Authors:** Mattias Myrenås, Karl Pedersen, Ulrika Windahl

**Affiliations:** 1 Swedish Veterinary Agency, Ulls väg 2b, SE-75189 Uppsala, Sweden; 2 Department of Animal and Veterinary Science, Aarhus University, Blichers Allé 20, 8830 Tjele, Denmark; karl.pedersen@anivet.au.dk

**Keywords:** MRSP, clonal lineages, canine, dog, multidrug-resistant staphylococci, *SCCmec*, whole-genome sequencing

## Abstract

Background/Objectives: *Staphylococcus pseudintermedius* is part of the normal microbiota in dogs. Since 2006, an increase in multidrug-resistant clones of methicillin-resistant *S. pseudintermedius* has been reported, as well as zoonotic transmission. Longitudinal investigations into clonal population structures, antibiotic resistance patterns, and the presence of resistance and virulence genes are important tools for gaining knowledge of the mechanisms behind the emergence of such clones. Methods: We investigated 87% of all non-repetitive MRSP isolates from dogs and cats in Sweden over a ten-year period (n = 356). All isolates were subjected to staphylococcal chromosomal cassette *mec* identification, whole-genome sequencing, multi-locus sequence typing, and analyses of genomic relatedness, as well as investigation of phenotypical resistance patterns and the presence of antibiotic resistance genes and virulence genes. Results: A considerable increase over time in the number of clonal lineages present was observed, indicating genomic diversification, and four clones became dominant: ST71, ST258, ST265, and ST551. In total, 96% of the isolates were multidrug-resistant. Statistically significant differences in resistance to several antibiotic classes between the four dominant clones were present. All isolates carried several virulence genes encoding factors associated with attachment, colonization, toxin synthesis, quorum sensing, antibiotic resistance, and immune evasion.

## 1. Introduction

The bacterial species *Staphylococcus pseudintermedius* (*S. pseudintermedius*) belongs to the *Staphylococcus intermedius* group (SIG), together with *Staphylococcus intermedius*, *Staphylococcus delphini*, *Staphyloccoccus cornubiensis*, and *Staphylococcus ursi* [1,2,3]. The core genomes of these species are related close to the threshold of species delineation but differ in properties that reflect the distinct ecological niches that each species occupies [1]. *Staphylococcus pseudintermedius* is a commensal microorganism in dogs as well as a well-known pathogen of major importance in dogs. It is associated with infections in a variety of body sites in dogs, but primarily with dermatologic infections such as pyoderma, otitis externa, and wound infections, from which it is the most commonly isolated pathogen [4,5]. Carriage of several *S. pseudintermedius* strains simultaneously was reported as early as in the 1990s [6]. Reports on staphylococcal carriage and infections in cats are less frequently published, but both carriage and infections with *S. pseudintermedius* are well known to occur, as is human colonization and infection, although the latter is less commonly reported [7,8,9,10,11]. One possible factor lowering the number of reports of zoonotic transfer and infection with *S. pseudintermedius* in humans is the misidentification of *S. pseudintermedius* as *Staphylococcus aureus* (*S. aureus*) due to lack of typing of coagulase positive staphylococci at the species level in human medicine [11,12,13].

In 2006, methicillin-resistant *S. pseudintermedius* (MRSP) emerged in canine populations in both North America and Europe [14]. Methicillin resistance is mediated by the *mec*A gene, which encodes the penicillin-binding protein 2A, conferring resistance to methicillin and other beta-lactam antibiotics. (PBP2a) [15]. In addition, MRSP isolates have generally been reported to have a multidrug-resistant (MDR) phenotype, i.e., the bacteria have acquired non-susceptibility to at least one agent in three or more antibiotic classes [4,9,16,17,18]. Since then, an increase over time in the proportion of MRSP relative to methicillin susceptible *S. pseudintermedius* (MSSP) in dogs with clinical infection has been reported from several countries. For example, Penna et al. (2022) reported that 12% of *S. pseudintermedius* isolates from Rio de Janeiro, Brazil, were methicillin-resistant, while Nocera et al. (2020) found that 23 out of 126 (18%) clinical *S. pseudintermedius* isolates from Portugal were methicillin-resistant [19,20]. From Finland, Grönthal et al. (2017) reported that 14% of clinical *S. pseudintermedius* isolates from dogs were methicillin-resistant [21]. An even higher proportion was reported from the United States, where 164 out of 200 *S. pseudintermedius* isolates from 2021 were confirmed to be MRSP (82%) [22]. The first MRSP clone appearing in Europe was the ST71 clone, detected in several European countries in 2006 [14,19]. The ST71 clone was subsequently also reported from both North and South America [14,19]. However, over time, several different clones have been identified and reported, primarily from Europe, North America, and Asia [22]. The mechanisms behind changes in clonal populations are so far poorly understood, as is the possible role of transmission of clones across national borders and within respective country.

The first case of MRSP infection in dogs in Sweden was reported in 2006, i.e., at the same time as MRSP was reported from other countries [23]. A further 11 cases were diagnosed in the same year. The number of cases increased over the following three years, and in 2009 a total of 130 cases were reported. From 2006 to 2009, national recommendations aimed at reducing the prevalence of antibiotic-resistant bacteria in pets were introduced, and the vast majority of Swedish veterinary hospitals and clinics quickly implemented these [24]. The recommendations included enhanced infection prevention and control, continued routine sampling and bacterial culture with antibiotic susceptibility testing of most bacterial infections in dogs and cats, as well as policies aimed at lowering the unnecessary use of antibiotics [24]. Despite this nationwide effort and subsequent regulations on infection prevention and control in veterinary care facilities and other similar dog-related business operations, as well as on preventive measures aimed at reducing the risk of community-acquired MRSP carriage and infections, MRSP infections still occur in the dog population. However, during 2010 and 2011 the number of such cases decreased and since then a relatively stable number of cases has been reported with a span of approximately 40 to 60 clinical cases of canine MRSP infections each year [24].

In addition to MRSP isolates being resistant to all beta-lactam antibiotics due to the acquisition of the *mec*A gene, resistance to several other antibiotic classes has often been reported, particularly aminoglycosides, macrolides, quinolones, sulphonamides, and tetracyclines [5,10,11]. The worldwide recognition of the appearance, spread, and increase in prevalence of MRSP clones is of great concern. It poses a challenge to both veterinary and human medicine due to zoonotic transfer leading to antibiotic-resistant infections not only in animals but also in humans [5,10,11].

There is a need for a better understanding of the mechanisms behind the emergence of antibiotic-resistant bacterial clones, including of the transmission of clones within and between countries versus independent genetic events leading to the establishment in the short or long term of new clones and new antibiotic resistance patterns. Investigations into changes in clonal population structure and phenotypical and genotypical antibiotic resistance patterns over time are important to better understand and predict the epidemiology and for the development of effective preventive measures aimed at preventing a further increase in the prevalence of MDR bacteria in veterinary and human healthcare. Furthermore, there is a relative lack of comprehensive investigations regarding presence of resistance genes related to phenotypic resistance as well as virulence genes in MRSP isolates and clones. The aims of this study were to investigate the population structure of MRSP, including possible changes over time, during a ten-year period and to investigate and present data on both antimicrobial resistance patterns and the presence of antibiotic resistance genes, as well as presence of virulence genes in a nationwide collection of strains from dogs and cats.

## 2. Results

### 2.1. Phenotypic Antibiotic Resistance Profiles

The results of the susceptibility testing and the classification of the 356 MRSP isolates as either wildtype (susceptible) or non-wildtype (resistant) are presented in Table 1. With the exception of nitrofurantoin, to which only one isolate was resistant, resistance to each of the antibiotic substances investigated was common. The lowest and highest percentages of isolates resistant to respective antibiotic, other than nitrofurantoin, were 61.6% (enrofloxacin) and 91.2% (erythromycin), respectively, as shown in Table 1. Furthermore, the majority (n = 341/356; 95.8%) of the isolates were multidrug-resistant (MDR), i.e., with acquired non-susceptibility to at least one agent in three or more antibiotic categories. The 15 isolates that were not MDR did not belong to any of the major STs but were mostly single isolates of an ST. Four isolates belonging to four different STs (ST731, ST1179, ST1384, and ST1390) were resistant only to beta-lactams, while seven isolates belonging to six different STs (ST550, ST1392, ST1388, ST1626, two ST1627, and ST2342) were resistant to beta-lactams and macrolides/lincosamides. One isolate (ST1624) was resistant to beta-lactams and macrolides but not to lincosamides, while one isolate was resistant to beta-lactams and fucidic acid (ST305), and two isolates (ST2122 and ST2352) were resistant to beta-lactams and tetracyclines. Most of the MDR isolates were also MDR even when excluding the beta-lactam resistance present in all the isolates.

### 2.2. Sequence Type Distribution

**Figure 1:**
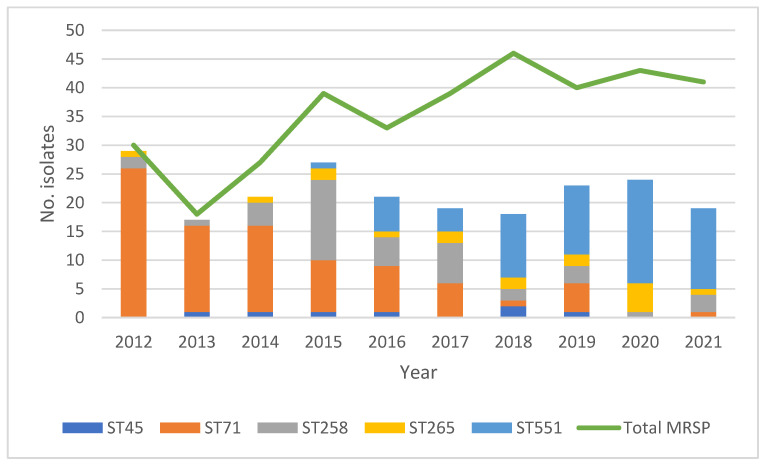
Distributions of both the total number of MRSP isolates per year *^1^ and the number of isolates belonging to the five STs represented by more than five isolates *^2^. *^1^ Total number of MRSP yearly: 2012: n = 30; 2013: n = 18; 2014: n = 27; 2015; n = 39; 2016: n = 33; 2017: n = 39; 2018: n = 46; 2019: n = 40; 2020: n = 43; 2021: n = 41. *^2^ Total number of isolates in respective ST over the ten-year study period: ST45: n = 7; ST71: n = 86; ST258: n = 42; ST265: n = 17; ST551: n = 66.

A total of 108 sequence types (STs) were represented, 95% (n = 103) of which were represented by only a single isolate (Supplementary Figure S1), leaving in total five STs represented by five or more isolates: ST45 (n = 7), ST71 (n = 86), ST258 (n = 42), ST265 (n = 17), and ST551 (n = 66). The distribution of these five STs is shown in Figure 1, together with the yearly number of clinical, non-repetitive cases of MRSP infections in dogs. Several notable changes in the relative prevalence of the respective STs over time are apparent.

In short, during the first three years (2012–2014), ST71 was the dominant type. During the following three years (2015–2017), ST71 continued to be dominant, but from 2015 the relative prevalence of isolates belonging to ST258 increased markedly, as did the relative prevalence of isolates belonging to ST45 from the year 2016 and onward. From 2018 and onward, ST551 was the dominant ST. Furthermore, a diversification of STs over time is notable. During 2012, 2013, and 2014, most isolates belonged to either ST71 or ST258. The number of STs represented by only one or a few isolates gradually increased from the year 2014 and onward, as did the number of STs represented by five or more isolates.

### 2.3. Antibiotic Resistance Amongst Represented Sequence Types

The percentage of non-wildtype isolates within each of the five most prevalent STs is shown in Table 2. In short, the proportion of isolates belonging to respective ST resistant to antibiotics other than nitrofurantoin and fucidic acid mainly ranged from 71 to 100%, with the notable exception of tetracycline resistance in isolates belonging to ST71 (16.3%), gentamicin resistance within ST258 (5%), and enrofloxacin resistance within ST258 and ST265 (0% and 7%, respectively) (Table 2). Several differences between the five most prevalent STs regarding the proportion of isolates resistant to specific antibiotic substances were statistically significant. This included enrofloxacin, to which all isolates typed as either ST71 or ST551, and 83% of isolates typed as ST45, were resistant but none of the 42 isolates in ST258, and only one (7%) of the in total 15 tested isolates in ST265. Likewise, for gentamicin, ST258 isolates were significantly less often resistant (5%) compared to isolates belonging to other STs, where either all, or 95% in ST71, were resistant. Furthermore, ST71 isolates were most often susceptible to tetracycline, with only 16% being of non-wild type, compared to 86–100% of isolates belonging to the other STs. This is illustrated in Table 2, where the percentages within each row that do not have a superscript letter in common are statistically different. For example, the proportion of isolates resistant to clindamycin in ST71 was statistically different when compared to the proportion of isolates resistant to clindamycin in ST258, but there was no such statistical difference when compared to the proportion of isolates resistant to clindamycin in other STs.

### 2.4. Genomic Relatedness of Isolates

**Figure 2:**
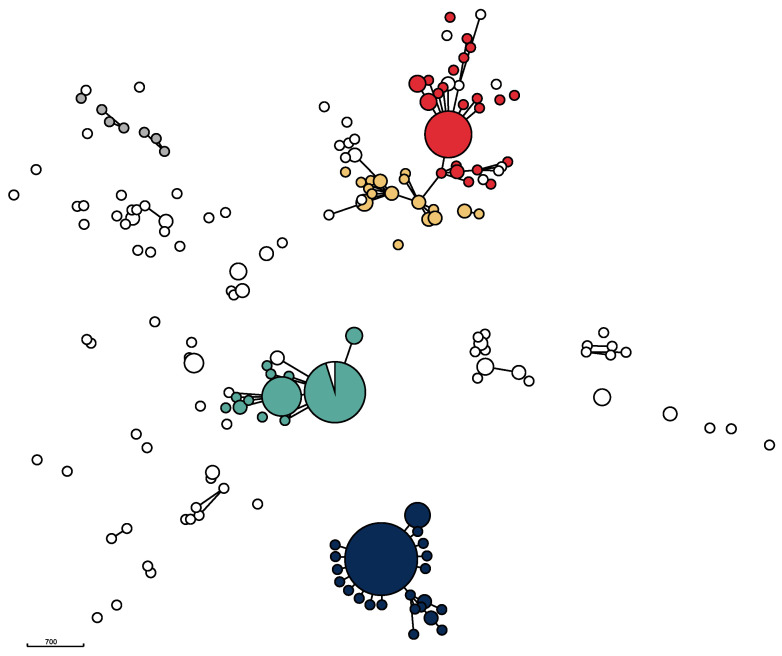
Minimum spanning tree for 356 Swedish MRSP isolates from dogs and cats. The five most prevalent STs are shown in the following colors: ST71: dark blue; ST551: green; ST258: red; ST265: orange; and ST45: grey. The MST is based on 2372 loci from wgMLST data. Isolates differing in 40 or less loci are grouped together. Disc size corresponds to the number of isolates. Isolates differing in 400 or less loci are connected with solid lines.

The genomic relatedness of the isolates based on the results of wgMLST data was visualized in a minimum spanning tree (Figure 2) as well as in a neighbor-joining tree (Supplementary Figure S1). The phylogenetic trees revealed considerable diversity, but also the presence of five major clusters centered around the five STs ST71, ST258, ST265, ST551, and ST45, as was several additional, minor clusters (Figure 2).

All ST71 isolates from the study period clustered closely together, indicating that the isolates with this ST remained essentially unchanged over the ten-year study period. The ST71 cluster was also the largest. It also included one ST358 isolate, which is a part of the CC71 complex. The majority of isolates belonging to ST551 formed a homogeneous, closely related group similar to the clustering of ST71-isolates. However, compared to the ST71 cluster, the ST551 cluster was more diverse. A number of other STs—ST1095, ST1338, ST2119, ST2269, and ST2270—also clustered with ST551 isolates. These isolates share five or six out of seven MLST loci and therefore belong to the CC551 complex. The third cluster, mainly containing ST258 isolates, also consisted of a core group of closely related isolates as well as of a number of less closely related isolates, including several different STs. The fourth major cluster contained ST265 isolates and related STs. Unlike ST71, ST258, and ST551, this cluster did not have a core of closely related isolates. Compared to the other three clusters, it was more diverse, containing several other STs. In addition to these four major clusters, several small clusters containing less than ten (ranging from two to nine) isolates were identifiable. For example, four ST181 isolates were clearly closely related to each other (Supplementary Figure S1). Likewise, three ST1627 isolates and three ST1331 isolates each formed small, closely related clusters, unrelated to other isolates. Furthermore, a group consisting of the seven ST45 isolates together with one ST282 and one ST1194 isolate, respectively, formed a heterogeneous subcluster.

### 2.5. SCCmec Identification and Distribution

In 98.3% (n = 350) of the sequences from the 356 isolates, either SCC*mec* elements or putative SCC*mec* elements could be characterized. A variety of SCC*mec* types were detected (Supplementary Figure S2). The distribution of different SCC*mec* elements was roughly similar to the population patterns as visualized in the neighbor-joining tree (Supplementary Figure S2). Only one of the 86 isolates belonging to the dominant ST71 cluster carried SCC*mec* Vc(5C2&5). The other 85 isolates carried the SCC*mec* II-III hybrid. This type was also carried by three other isolates, of which one was ST358, closely related to the ST71 isolates. The remaining two isolates carrying the SCC*mec* II-III hybrid were more distantly related to the ST71 isolates and belonged to ST826 and ST2123, respectively. In total, 92 isolates carried SCC*mec* Vc(5C2&5) isolates, including all 66 isolates belonging to the ST551 cluster. Of the remaining 26 isolates carrying SCC*mec* Vc(5C2&5), only ten were related to the ST551 isolates. The *czrC* gene was detected in all SCC*mec* Vc(5C2&5) but not in any other SCC*mec.* Another SCC*mec* type, SCC*mec* IVg, was carried by isolates within the branch of the phylogenetic tree containing ST265, ST258, and related STs (n = 94) (Supplementary Figure S2) as well as in 11 more distantly related isolates. Yet another element, the pseudo-ΨSCC*mec*_57395,_ was detected in all seven ST45 isolates as well as in three related STs clustering together. In addition, other SCC*mec* element types were detected (Supplementary Figure S2). When illustrating the association between STs by constructing a minimum spanning tree (Figure 2), the three STs ST71, ST551, and ST45 grouped separately, whereas ST258 and ST265, which shared aSCC*mec* type, grouped together.

### 2.6. Carriage of Antibiotic Resistance Genes

A total of 21 antibiotic resistance genes and two point mutations conferring antibiotic resistance were detected (Table 3). Nine of the genes and both point mutations were present in more than half of the isolates (Table 3, Supplementary Figure S1).

#### 2.6.1. Beta-Lactam Resistance Genes

All isolates carried the *mecA* gene, which confers resistance to methicillin and other beta-lactam antibiotics. In addition, 96% (n = 343) of isolates carried a *blaZ* gene, which encodes a beta-lactamase. No other beta-lactam resistance genes were detected.

#### 2.6.2. Aminoglycoside and Aminocyclitol Resistance Genes

Several genes conferring resistance to antibiotics of the aminoglycoside group were present, and in most isolates more than one gene was present. The high level of gentamicin resistance can, in 95.3% of the gentamicin-resistant isolates, be attributed to the presence of *aac(6′)-Ie/aph(2″)-Ia*. We did not test for phenotypic resistance to neomycin or streptomycin, but *aph(3′)-IIIa* conferring resistance to both compounds was present in 318 (89.3%) isolates. Resistance to streptomycin may also be caused by either *spw* or *ant(9)-Ia*. In 38 (10.7%) of isolates, *spw* was present and *ant(9)-Ia* was present in two (0.6%) isolates. No isolates carried both genes. The *sat4* gene, which confers resistance to streptothricin, was present in the majority of isolates (79.2%).

#### 2.6.3. Macrolide and Lincosamide Resistance Genes

The *erm*(B) gene was present in 89.0% of the isolates (n = 317), all of which were phenotypically resistant to erythromycin. Notably, an additional ten isolates were phenotypically resistant to erythromycin, despite not carrying the *erm* gene. The *erm*(A) gene was detected in only three isolates. *lnu*(A) and *lnu*(B), which confer resistance to lincosamides but not to macrolides, were present in three (0.8%) and 38 (10.7%) isolates, respectively. *lnu*(G) was not detected. The *lsa*(E) gene, which confers resistance to streptogramins, lincosamides, and pleuromutilins, was detected in 38 isolates (10.7%). These 38 isolates were the same as the 38 isolates carrying *lnu*(B) and the *spw* genes.

#### 2.6.4. Amphenicol Resistance Genes

Two genes conferring resistance to amphenicols were detected: the *catA* gene, which confers resistance only to non-fluorinated amphenicols in 23.3% of all isolates (n = 83), and *fexA*, which confers resistance to both chloramphenicol and florfenicol in 0.8% of all isolates (n = 3), with no isolate having both genes.

#### 2.6.5. Tetracycline Resistance Genes

Three different tetracycline resistance genes were detected: *tet*(K), *tet*(L), and *tet*(M), with *tet*(M) being the most prevalent (60.7%, n = 216) followed by *tet(K)* (22.8%, n = 81) and *tet(L)* (1.4%, n = 5). Phenotypical resistance to tetracycline was present in all isolates carrying one or more of the three genes in question.

#### 2.6.6. Folate Pathway Inhibitor-Associated Genes

The *dfrG* gene was detected in 81.1% of all isolates. No other resistance genes known to confer resistance to sulphonamides were detected.

#### 2.6.7. Fluoroquinolone Resistance Genes and Mutations

Point mutations in the genes *grlA* (Asp84Gly/Asn/Tyr, Ser80Ile/Arg) and *gyrA* (Ser84Leu), all of which confer resistance to fluoroquinolones, were present in 63.6% (n = 222) and 57.8% (n = 206) of the 356 isolates, respectively. The presence of point mutations in both genes was commonly occurring, with the combination of *grlA*:Ser80Ile and *gyrA*:Ser84Leu being the most common (56.2%, n = 200). Three isolates only had the*gyrA* mutation. All three isolates had MIC values ≤ 0.25 µg/mL for enrofloxacin, i.e., this mutation alone did not cause phenotypic resistance. Nineteen isolates carried all of the searched for mutations in the *grlA* gene, but not the searched for mutation in the *gyrA* gene. In seven of these 19 isolates, the MIC values for enrofloxacin were higher than the ECOFF 0.5 µg/mL, i.e., these isolates were phenotypically resistant, while two isolates were not tested for enrofloxacin. All isolates in which mutations were detected in both genes were phenotypically resistant to enrofloxacin.

### 2.7. Stress Response-Associated Genes

In addition to the antibiotic resistance genes, 18 isolates also carried genes associated with resistance to quaternary ammonia compounds and chlorhexidine. Sixteen isolates carried the *qacG* gene and two isolates carried the *qacJ* gene. We did not test for susceptibility to these compounds.

The *czrC* gene conferring cadmium and zinc resistance was detected in 93 isolates (26.1%), all carrying *SCCmec* Vc(5C2&5), i.e., belonging to the ST551 cluster (Supplementary Figure S2).

### 2.8. Toxin and Virulence Genes

All isolates harbored a long array of virulence genes (Supplementary Figure S2). These virulence factors can be grouped into five categories: as genes encoding for cell communication, adhesion to and colonization of host cells and tissue, invasion and damage of host cells and tissues, or for immune evasion; genes encoding for cell communication; those encoding for host cell and tissue adhesion and colonization; those encoding for host cell and tissue invasion and damage; and those encoding for immune evasion.

All isolates carried the genes *agrA*, *agrB*, *agrC*, and *agrD*, which encode for quorum sensing, a communication system between bacteria.

For adhesion and colonization, all the isolates carried several *Staphylococcus pseudintermedius* surface protein genes (*spsA*–*spsR*), which encode for attachment to for example collagen, fibrinogen, fibronectin, or cytokeratin. The presence of the *nanB* gene, which encodes for a neuraminidase B enzyme, was variable but in general absent in ST45 and ST71 isolates and present in ST258 and ST265. No isolates carried the genes *clfA*, *clfB* (clumping factor A and B), *fnbA*, or *fnbB* (fibronectin-binding proteins A and B).

All isolates carried genes encoding several toxins: gamma-haemolysin B, *hlgB*, the leucotoxins *lukF* and *lukS* encoding the Panton-Valentine leukocidin (PVL); phenol-soluble modulins, *psmA*, *psmB*, *psmD* and *psmE*; the enterotoxins *sec-Canine*; the exfoliative toxin, *siet*; and the nuclease *nucC*. Notably, no isolates carried the gene *tst*, which encodes for staphyloccal toxic shock syndrome toxin (TSST-1).

The *S. pseudintermedius* surface proteins *spsA*, *spsB*, *spsE*, *spsG*, and *spsN* genes were present in all isolates and *spsC* and *spsH* in all except one, whereas *spsF*, *spsJ*, and *spsO* were absent in all of the isolates.

For *spsD*, *spsI*, *spsK*, *spsL*, *spsM*, *spsQ*, and *spsR*, presence was variable, although for *spsK* most ST551 had the gene and most others did not, and for *spsL*, most ST551 had the gene and most others did not.

For immune evasion, all isolates carried genes encoding biofilm formation: *icaA*, *icaB*, *icaC*, and *icaD*, and for coagulase, *coa*.

## 3. Discussion

The material included in this study is unusual, as due to national regulations, it includes 87% (n = 356) of all MRSP isolates recovered from dogs and cats in the country over a ten-year period, i.e., a representative national selection of clinical isolates. This allowed for a linear time study, which showed changes in the population structure of MRSP clones over time in a defined geographical area. Our data show that, over time, both a clear diversification with the appearance of new STs and a change in the relative prevalence of four dominant clones took place. The number of STs increased from the year 2014 and onward, leading to over a hundred STs being present during the study period. Interestingly, the vast majority (95%) of STs were represented by only one single isolate each in the dataset.

The first clone to appear in Sweden was ST71, in the year 2006, and it remained the dominant clone until 2018 [24]. This result correlates with other publications, including by Perreten et al., (2010) who investigated 103 MRSP isolates stored in both European and North American laboratories from the year 2004 to 2009 and found that, in Europe, ST71 was the dominant clone during that time [14]. However, a diminishing relative prevalence of the ST71 clone over time after its first appearance in Europe in 2006, as well as a diversification in the population structure of MRSP clones, some of which may belong to the CC71 clonal complex, have been reported from other European countries as well [17,18,20,21,22,25]. Papić et al. (2021) investigated 43 MRSP isolates collected from five Slovenian small animal clinics during 2008–2018 and found that although most of the isolates belonged to ST71, a second clone, ST551, appeared during the last three years of the study [25]. Nocera et al. (2020) investigated 126 MRSP isolates collected from canine dermatology cases in Naples and the Latina province of Italy during 2015–2017 and although 26% of the isolates belonged to ST71, nine new sequence types were described for the first time and were named ST1053–ST1061 [20]. Silva et al. (2021) investigated 31 isolates collected during 2019 from canine pyoderma cases in Portugal [18]. In a Finnish study, Grönthal et al. (2014) investigated 266 MRSP isolates collected from companion animals over a 4.5-year period (2011–2015) and found that, over time, the CC71 clone was gradually displaced first by CC45 and CC258 and subsequently by other clonal lineages as well [26]. In a Dutch study, three major clonal lineages were found among 50 canine isolates collected in 2004 in Utrecht: CC71, CC258, and CC45 [17]. In addition, a recent North American investigation of MRSP isolates collected in the United States compared the population structures of 141 MRSP isolates from 2010 with those of 164 isolates from 2021 [22]. In 2010, over one third of the isolates (35.7%) belonged to ST68, making it the dominant clone, followed by ST84 and ST71. This had changed by 2021, with the most common STs being ST45, ST155, ST181, ST496, and ST551 [22].

Four STs were dominant in our study: ST71, ST551, ST258, and ST265. These STs have been described in other European-based studies as well, including by Wegener et al. (2018) and Phophi et al. (2023) [17,22]. However, interestingly, although ST551 was one of the four dominant clones in Sweden during the study period, represented by 66 isolates, other published European reports in which ST551 is mentioned are few. In a study by Bergot et al. (2018), only one single isolate was detected [27]. In contrast, Phophi et al. (2023) reported this ST as one of the dominant types in the United States [22]. It is unknown how this clone was introduced and became the most recent dominant clone in Sweden. Notably, no isolates belonging to ST68 were present in our study material.

We found a considerable diversity among the isolates, with 110 different STs represented, the presence of several small clusters unrelated to others and lacking a common recent ancestor, as well as a variety in SCC*mec* types. These findings indicate a high genomic plasticity of MRSP and suggest that at least some MRSP clones may have evolved independently on several occasions. Similar results were reported in a study by Phophi et al. (2023), in which several of the nine STs described for the first time did not seem to share any recent ancestor with the dominant types from 2010 [22]. The phenomenon of clonal displacement has been reported for other bacteria as well, including methicillin-resistant *S. aureus* (MRSA) and *Salmonella*. For example, MRSA CC398 spread rapidly throughout the entire European pig population [28]. Furthermore, a monophasic *Salmonella* Typhimurium 4,[5],12 has become dominant in Europe [29]. However, overall, the mechanisms behind clonal successions such as these are currently essentially unknown, as is the possible role of clonal transmission across national borders and within respective countries through direct and indirect transmission from infected dogs to other individuals.

On the other hand, some of the STs in our study were highly conserved (Figure 2, Supplementary Figure S1). This was most prominent for ST71, the first MRSP clone described in Europe, including in several European countries over a rather short time period [14]. During our study period, this clone hardly deviated genomically. In contrast, the ST45 and ST265 clusters were highly diverse and contained other STs as well. The ST258 and ST551 clusters were in between, both having a core group of highly conserved isolates as well as a several genomically deviating isolates. The reasons for this apparent highly conserved population structure of certain STs and highly variable population structure of others is not known and warrants further investigation.

Interestingly, the ST45 ΨSCC*mec*_57395_ detected in our study was in 2013 reported to be the predominant clone in Thailand and Israel [30]. The possibility of a dog entering Sweden carrying such an MRSP strain cannot be excluded. Another possibility is that genetic changes have occurred within Sweden, independent from strains in other countries. Further investigations into the relative importance of genetic adaptation to antibiotic pressure versus the importance of direct transmission of MRSP strains between dogs for the spread and increase in prevalence of MRSP as well as for changes in clonal population structure are warranted. The variety of STs and SCC*mec* elements in the study material suggests that new clones of MRSP have emerged independently of each other. On the other hand, it is to be expected that the increased prevalence of at least some of these clones is due to direct and indirect transmission of MRSP isolates from dogs, both within and across national borders, as such transmission has been shown to occur within veterinary health care settings as well as in households [8]. This was further elucidated in a previous investigation by Windahl et al. (2012), where it was concluded that dogs diagnosed with clinical MRSP infections can continue to be carriers of the bacterium for more than one year, and that systemic antibiotic treatment may prolong the carrier period [31]. Notably, though, another study by Windahl et al. (2016) showed that contact dogs living in the same household as a dog with an MRSP infection did not necessarily become MRSP carriers [32]. Identified risk factors for MRSP carriage and infections in dogs include antibiotic treatment, particularly long-term treatment [31,33], and treatment in veterinary healthcare settings, such as hospitalization [34]. Furthermore, outbreaks of MRSP infections have occurred in veterinary clinic settings, similar to MRSA outbreaks in human healthcare settings, highlighting the need for proper hygiene precautions in veterinary hospitals, including hand hygiene and disinfection of contact surfaces [26,35]. We do not have data on to which extent the sampled dogs had been exposed to such risk factors, or whether some of the dogs had been in contact with each other. This warrants further investigation, and we hope to be able to perform such a study in the future.

We could determine SCC*mec* elements or putative SCC*mec* elements in the majority (98.3%) of the 356 investigated MRSP isolates. In the remaining six isolates, either an SCC*mec* element could not be found, or the result of the typing was deemed to be ambiguous. Five of these were unclustered, single STs unrelated to other isolates. It would be interesting to investigate the genomic location of their *mecA* gene in further detail. The sixth isolate, belonging to ST1296, was very closely related to another ST1296, which had an ΨSCC*mec*_57395._ Furthermore, both of these ST1296 isolates were related, although not closely, to two other isolates carrying the same SCC*mec*. These four isolates were in turn unrelated to the ST45 cluster with the same SCC*mec*, indicating that they had acquired the SCC*mec* through a different genomic event.

Interestingly, although most of the ST71 isolates carried SCC*mec* II-III, one ST71 isolate that was more distantly related to the others carried SCC*mec* Vc(5C2&5) with *czrC.* This may suggest a separate genomic event replacing the SCC*mec* element. The *czrC* gene, which confers resistance to zinc and cadmium, was detected in all isolates with SCC*mec* Vc(5C2&5), i.e., the ST71 isolate mentioned above and the isolates belonging to ST551 and related STs (Supplementary Figure S2).

We found one previous report describing the *czrC* gene in MRSP [36]. The authors found the gene in two MRSP isolates from Argentina, both with SCC*mec* Vc(5C2&5), but the ST was not revealed [36]. Therefore, our finding of *czrC* in all the ST551 isolates as well as in related STs was surprising. The gene is mainly associated with MRSA CC398, which also often carries SCC*mec* Vc(5C2&5) with the *czrC* gene located inside the SCC*mec* cassette [36,37,38,39]. The *czrC* gene is often present in MRSA CC398 isolates from pigs, and it is assumed that its presence is facilitated by the use of high-level zinc oxide in pig feed for the prevention of postweaning diarrhoea, thereby giving these strains an advantage compared to strains without *czrC* [40]. In contrast, MRSA CC398 from humans rarely carry *czrC*, probably because it has been lost due to the lack of any selective pressure [37]. It is uncertain what the selective advantage of the gene is for an MRSP strain, and it may be coincidental due to the presence of this gene by acquisition of the SCC*mec* cassette.

McCarthy et al. (2015) reported that the emergence of a multidrug-resistant MRSP requires a three-step process, and that this may occur quite easily [41]. An SCC*mec* element may be adopted from other staphylococci, such as *S. aureus*, but in the case of the SCC*mec* II-III hybrid, one element comes from *S. epidermidis*, and for the ΨSCC*mec*_57395_, one element comes from *S. haemolyticus* [41]. We believe that long-read sequence data could give more insights into the SCC*mec* elements in our material and hope to further pursue such investigations. Antibiotic resistance was highly prevalent to almost all antibiotics tested in our study. In addition to all MRSP isolates being resistant to beta-lactams, the vast majority (95.8%) were multidrug-resistant. The percentages of isolates resistant to these antibiotics ranged from approximately 60–70% (enrofloxacin, 62%; tetracycline, 67%; gentamicin, 72%) to approximately 86–90% (sulphonamide–trimethoprim, 86%; clindamycin, 87%; erythromycin, 92%). This is a cause for concern regarding treatment options, not only for companion animals but also for human beings when zoonotic transfer occurs [10,11]. Notably, this overall resistance pattern differs fundamentally from that recorded for MSSP in Sweden, where resistance to any of the antibiotic classes is relatively uncommon, with approximately one fifth of the isolates being susceptible to all of these antibiotics [24]. Furthermore, the proportion of MSSP isolates resistant to five or more classes of antibiotics, including beta-lactams, has halved since 2016, from almost one third to 15% [24]. For example, the proportion of Swedish MSSP isolates resistant to clindamycin and tetracycline is currently approximately 15% for each compound [24]. Furthermore, although enrofloxacin resistance was seen in a lower percentage among the MRSP isolates compared to other compounds, it was still 63%, compared to less than 1% of Swedish MSSP isolates [24]. The prevalence and risk of genetic transfer of antibiotic resistance from MRSP isolates to MSSP isolates warrants further investigation, together with further investigations into the mechanisms and the frequency of changes in the genetic makeup of MRSP and MRSA clones. This includes genetic events within the interface between different animal species, as well as between humans and animals. Dogs living as pets in human households are of particular interest, as they live so close to humans, with subsequent possibilities of interspecies transfer of relevant microbiota and resistance genes. The need for further investigations into the importance of and underlying mechanisms driving genetic events leading to the development of antimicrobial resistant clones is also highlighted by the relatively stable number of MRSP cases reported in Sweden over the last decade. The presence of MRSP infections and the development of new clonal lineages have not declined despite a steady decline in the overall sales of veterinary medicinal antibiotic products for oral medication of dogs leading to a 76% reduction in sales from the year 2006 to 2023. This includes a reduction in sales of cephalosporins (−90%), fluoroquinolones (−95%), and aminopenicillins with clavulanic acid (−88%). As mentioned above, the reduction in the use of antibiotics is part of a bundle of policies aimed at reducing the prevalence of antibiotic-resistant bacteria in pets that has been implemented by the vast majority of Swedish veterinary hospitals and clinics, including enhanced preventive infection control measures.

In the presentstudy, several statistically significant differences in the proportions of resistant isolates among the five most prevalent STs were detected regarding prevalence of resistance. These were most evident for enrofloxacin, gentamicin, and tetracycline. When comparing the phenotypical resistance patterns to the presence of antibiotic resistance genes, such differences could generally be attributed to the presence or absence of certain resistance genes or mutations. However, there was not a complete correspondence between phenotypic and genotypic resistance for all compounds and isolates. Bergot et al. (2018) also reported differences in resistance patterns between STs [27]. Notably, in the study by Bergot et al., isolates belonging to ST71 had higher resistance levels to almost all antibiotics compared to non-ST71 isolates, whereas in our study the highest resistance levels, for most compounds 100%, were recorded for ST551, the currently most dominant type in Sweden.

A high occurrence of the *blaZ* in the MRSP isolates such as in our study (96.3%) has also been reported in other studies on antibiotic resistance genes in MRSP isolates [14,17,18,42]. For comparison, approximately 70% of MSSP isolates from Swedish dogs tested by SVA during the year 2021 showed phenotypic resistance to penicillin due to penicillinase production, making it the most common resistance encountered in tested MSSP isolates, despite a decrease in the relative prevalence from 90% in the year 2009 [24]. Further investigations into the genetic makeup of MSSP over time would be of interest.

The presence of the different aminoglycoside resistance genes conferring resistance to different antibiotics from this heterogeneous group, such as streptomycin, kanamycin, gentamicin, tobramycin, neomycin, paromomycin, spectinomycin, and streptothricin, several of which were carried by most of the isolates, was reflected in the phenotypic results, where most of the isolates were resistant to gentamicin. Similar results were reported by Perreten et al. (2010) and by Silva et al. (2021), whereas Wegener et al. (2018) only reported the presence of the two genes *aac(6)-aph(2)* and *aph(3*′*)-III*, which confer gentamicin and kanamycin resistance [14,17,18].

The presence of the *erm*(B) gene could explain the high level of resistance to erythromycin as all except one of the 317 isolates carrying the *erm*(B) gene were phenotypically resistant to erythromycin. However, an additional ten isolates that were phenotypically resistant to erythromycin did not carry the *erm*(B) gene. It is possible that other mechanisms were the cause of the resistance present in these ten isolates, or alternatively that we failed to detect a macrolide resistance gene. All of the three isolates that carried the *erm*(A) gene also carried *erm*(B), thus it was not possible to evaluate the individual effect of the presence of *erm*(A).

The individual effect of the *lnu*(A) gene detected in three isolates, all resistant to clindamycin, cannot be evaluated, as one isolate also carried *lnu*(B) and the other two *erm*(B). Notably, *lnu*(B) was more common, as it was present in 38 isolates, whereas *lnu*(G) was not found.

The *lsa*(E) gene encodes for resistance to pleuromutilins, lincosamides, and streptogramins. It has been described to be located on plasmids in enterococci which can be conjugated into other bacteria [43]. Recent reports have also described this gene in streptococci and *S. aureus*, particularly in some isolates of MRSA CC398 from pigs, but also from ST9 isolates [44]. Furthermore, the gene has been detected in staphylococci from selective cultures of samples from healthy dogs and cats in China [45]. However, to the best of our knowledge, the present study is the first to report the presence of the *lsa*(E) resistance gene in *S. pseudintermedius*, as well as the first to report it in a canine sample. Interestingly, Wu et al. (2022) reported that the *lsa*(E) gene as located in multi-resistance gene clusters together with *spw* and *lnu*(B), and in our study all 38 isolates carrying the *lsa*(E) gene were also carrying *spw* and *lnu*(B) [46]. We plan to conduct further studies on the resistance gene environment in these 38 isolates.

Amphenicol resistance can be conferred by a number of genes. Several authors have reported the presence of *cat_pC221_* genes in MRSP, in isolates from both European countries and North America [14,17,42]. The genes *cat_pC221_* and *catA* confer resistance to chloramphenicol and the two *catA* accessions in the AMRFinder+ database share 97.2% and 98.1% amino acid similarities to *cat_pC221_*, respectively. In our study, *catA* was present in 23.3% of the isolates, with no discernible difference in the relative prevalence depending on the clonal lineage. We did not look for *cat_pC221_* and we did not include chloramphenicol in our test panel, but other researchers have reported that isolates harboring the *cat_pC221_* gene were indeed resistant to chloramphenicol [14,17,42]. Combined with the results of the present study, we therefore suspect that chloramphenicol resistance might be quite common in Swedish MRSP isolates, irrespective of clonal lineage.

The presence of the *fexA* gene, which confers resistance to both non-fluorinated (i.e., chloramphenicol) and fluorinated (i.e., florfenicol) amphenicols, has previously been reported by Schouls et al. (2022) together with a third amphenicol resistance gene, *cfr*, in MRSA isolates from the Netherlands, while a *fexA* variant, which did not confer florfenicol resistance, was described in a *S. pseudintermedius* isolate from a healthy dog in Spain by Gomez-Sanz et al. (2013) [47,48]. In another study conducted more recently in Spain, it was detected in two canine Staphylococcus isolates (MRSP and MSSP, respectively) with MIC values for florfenicol >16 µg/mL; [49]. We detected the *fexA* gene in three isolates, and we hope to be able to study these isolates further, concerning both the exact sequence of the gene and its impact on susceptibility to amphenicols.

Two of the three tetracycline resistance genes detected in our study (*tet*(K), *tet*(L) and *tet*(M) have been reported as commonly carried by MRSP isolates: *tet*(K) and *tet*(M) [14,17,18]. However, while *tet*(L) has been found in a number of different *Staphylococcus* species [50], we did not find any previous references to its occurrence in *S. pseudintermedius,* in neither MSSP nor MRSP isolates.

In total 86% of the 356 isolates in our study were classified as non-wild type to sulphonamide–trimethoprim, and 81% carried the *dfrG* gene, a gene encoding a dihydrofolate reductase enzyme which is resistant to the inhibitory effect of trimethoprim. Notably, no other gene known to confer trimethoprim resistance was detected, and no isolates carried any genes conferring sulphonamide resistance, which is common in many bacterial species, including in *Enterobacteriales*, encoded by *sul1*, *sul2*, or *sul3* genes [51]. The combined presence of *dfrG* and absence of sulphonamide resistance genes in MRSP isolates has, however, been reported by other researchers as well [14,17].

Resistance to fluoroquinolones is currently thought to most often be caused by point mutations in the genes encoding DNA gyrase (*gyrA*, *gyrB*) or topoisomerase IV (*grlA*, *grlB*). Other mechanisms include efflux pumps or disruption of interaction with fluoroquinolones by binding to topoisomerase, encoded by *qnr* genes [52]. In our study, 61.9% of the MRSP isolates were resistant to enrofloxacin, which can be compared to the significantly lower 1% in MSSP [24]. Furthermore, 57.0% of the investigated MRSP isolates had point mutations in both *gyrA* and *grlA*. Interestingly, the three isolates with only *gyrA* mutations were not phenotypically resistant, and of the 19 isolates with only *grlA* mutations, two were phenotypically resistant. In contrast, all isolates carrying point mutations in both genes were phenotypically resistant. A high prevalence of fluoroquinolone resistance in MRSP isolates compared to that of MSSP isolates has also been reported by other authors, as has the presence of point mutations in both genes [17,41]. Azzariti et al. (2022) reported that only 5.7% of investigated MSSA isolates from the United Kingdom were resistant to fluoroquinolones compared to 94.2% of the MRSP isolates [52]. An investigation of a subset of resistant isolates in their study revealed point mutations in both genes: a Ser84Leu mutation in *gyrA* and a Ser80Ile mutation in *grlA*, a combination of point mutations that we found in 56.2% of the isolates in the present study.

The use of disinfectants is widespread in veterinary clinics. Common compounds used include ethanol, chlorhexidine, and quaternary ammonium compounds, such as benzalkonium chloride. Importantly, chlorhexidine-based shampoos are commonly used as a long-term treatment in canine dermatology cases, and such products have been specifically suggested as a tool in decolonization of MRSP-carrying dogs [4,8]. It is therefore highly notable that in total 18 MRSP isolates carried *qac* genes, i.e., genes that confer resistance to quaternary ammonia compounds and may cause decreased susceptibility to chlorhexidine. However, we did not test for susceptibility to disinfectants and the isolates may still be susceptible despite the presence of *qac* genes. Several *qac* genes encoding for multidrug efflux pumps have been described in different bacterial species. In staphylococci, six different efflux pumps located on plasmids have been described [53]. The mechanisms leading to chlorhexidine resistance are not well understood, and the presence of *qac* genes does not necessarily lead to increased MIC values for chlorhexidine. Investigations of *qac* genes in staphylococci have most often concentrated on *S. aureus* in clinical settings with high prevalences reported from several countries [53]. The use of certain disinfectants in human health care settings has been proposed to possibly select for the presence of these genes in S. *aureus* and *Staphylococcus epidermidis* [53]. Staphylococci carrying *qac* genes have also been detected in food processing plants and an association with the use of disinfectants such as benzalkonium chloride in such production areas has been suggested [54]. Resistance to disinfectants may also facilitate persistence of MRSP strains in veterinary clinics, and systematic studies on presence of *qac* genes in *S. pseudintermedius* and their influence on MIC values and persistence in veterinary clinics are warranted. Furthermore, these results highlight the importance of strategic infection prevention in veterinary care facilities. There is an inherent risk for personnel to focus on which disinfectants should be used, and for personnel to rely too much on the use of such substances, as this is seen as less time-consuming and complicated than the cornerstones of infection control programs. Key principles that cannot be replaced by use of disinfectants include, for example, decreasing exposure through identification and isolation of risk patients regarding carriage of MDR bacteria, thereby avoiding transfer through direct contact, as well as measures to prevent indirect transfer through cleaning, washing of materials, and proper hand hygiene measures. Use of disinfectants alone cannot replace proper infection prevention, but it might contribute to the development of bacterial resistance, i.e., such substances should be used strategically and not habitually. Some, for example chlorhexidine-based shampoos, are also useful tools for treatment of dogs with resistant bacterial skin infection, as well as a tool used with the aim of decreasing the staphylococcal load. i.e., to decrease the risk of transfer of MDR bacteria, including MRSP and MRSA, to others. Bacterial resistance to such treatment could therefore hamper not only the quality of life for the individual, infected dog, but also lead to an increased risk of spread of MDR staphylococci.

All the isolates carried genes similar to the accessory gene regulator–system (*agr*) found in *S. aureus*. The *agr* system is a quorum-sensing and signal transduction system for communication between bacteria, which is important for the regulation of key elements of the infection process, such as biofilm formation, the expression of virulence factors, and the production of secondary metabolites [4,55].

The first step in the infection process is the attachment to host cells or, for example, connective tissue proteins such as collagen, fibrinogen, fibronectin, elastin, or keratin. The results of the whole-genome sequencing revealed that MRSP possesses an arsenal of genes encoding adhesion and colonization factors, such as microbial surface components recognizing adhesive matrix molecules (MSCRAMMs) [56]. These include *Staphylococcus pseudintermedius* surface protein genes, *spsA* through to *spsR*, of which all isolates carried several, although with differences in distribution and amount of such genes, a distribution pattern that to some extent was associated with ST-bases population patterns (Supplementary Table S2). These genes have also been reported to be present in MSSP isolates, which suggest that they are important in the infection process for *S. pseudintermedius* in general [57,58,59]. The MSCRAMMs *fnbA* and *fnbB*, coding for the fibronectin-binding proteins A and B, respectively, were not detected in our isolates, and neither were the MSCRAMMs *clfA* nor *clfB*, coding for clumping factors A and B, respectively.

Another gene involved in the adhesion and colonization process is *nanB*, which encodes a neuraminidase (sialidase), which cleaves terminal sialic acid residues from various glycolipids and glycoproteins associated with cell surfaces and body fluids. This may lead not only to damage to cell surfaces, but also to the unmasking of potential cell surface receptors for *S. pseudintermedius* [60,61]. Rynhoud et al. (2021) concluded that all MRSP isolates in their study carried a similar range of virulence genes; however, differences were observed for *nanB*, and these differences were associated with STs [60]. We also observed differences in the occurrence of this gene: most of the ST45 and ST71 isolates had the gene whereas most of the ST258 and ST265 isolates did not. Rynhoud et al. (2021) speculated that this could cause differences in virulence between STs [60]. This may well be the case, but our data do not provide the evidence to allow us to conclude this. The MRSP isolates additionally carried genes encoding for other enzymes: the two proteases *clpA* and *clpX*, which can assist in degrading host cells or cell components; coagulase (*coa*), which can assist in immune evasion; and nuclease (*nucC*), which can coat the surface of the bacterium with fibrin, which in turn protects against phagocytosis.

All isolates possessed the genes *icaA*–*icaD*, both of which are involved in biofilm formation, i.e., another immune evasion mechanism. This property is not unique for MRSP but seems to be common for *S. pseudintermedius*, as several researchers have reported that essentially all *S. pseudintermedius* carry these genes [58,59,62,63]. We did not investigate phenotypic formation of exopolysaccharide biofilm formation, but previous observations have suggested that only *icaA* and *icaD* are essential for exopolysaccharide synthesis [64].

All isolates had the potential to produce several toxins, exfoliative toxins, haemolysin, leucotoxins, phenol-soluble modulins, and enterotoxins. The two exfoliative toxin genes *siet* and *speta* were present in all isolates, while *expA* and *expB* occurred in only a few isolates. These findings seem to be in agreement with findings in other studies on *S. pseudintermedius*, although not all of them included all genes [58,59,62,63,65]. The *lukF-PV* and *lukS-PV* leucotoxins, which are equivalent to PVL in *S. aureus*, *hlgB* encoding for the gamma-haemolysin B component, and enterotoxin *sec-Canine,* were found in all the isolates, and also seem to be universal properties of *S. pseudintermedius* [58,59,62,63,65]. However, it is noteworthy that while Glajzner et al. (2023), Breyer et al. (2023), and Hritcu et al. (2020) found both *lukS* and *lukF* in essentially all investigated isolates, as we did in this study, Wang et al. (2022) did not find *lukF* in any of their isolates. It is possible that these differences are connected to the laboratory methods used [58,59,63,65].

In our study, all isolates carried the *psmA*, *psmB*, *psmD*, and *psmE* genes. To the best of our knowledge, this is the first reported occurrence of these specific genes in MRSP isolates. Variants of the genes have previously been detected in different staphylococcal species where they are known to encode for the so-called phenol-soluble modulins (PSMs) [66]. In *S. aureus*, they have been ascribed a variety of properties, such as cell lysis, inflammatory response stimulation, and biofilm formation [66]. Our finding ofthese genes in all of the investigated MRSP isolates indicate a need for further investigations into the prevalence and role of these as virulence factors in MRSP and MSSP.

## 4. Materials and Methods

### 4.1. Bacterial Isolates and Species Identification

The isolates investigated in the present study were non-repetitive isolates of canine or feline origin sampled in Sweden from 2012 to 2021 by veterinary clinicians. They were either submitted directly to SVA as clinical samples for bacterial culture, identification, and susceptibility testing, or as isolates as regulated by national authorities for confirmation of MRSP after culture and species identification performed at other laboratories had indicated possible presence of MRSP isolates in the clinical sample. All isolates identified as MRSP during the five-year period of 2017–2021 were included in the study. Due to financial restrictions, we were not able to perform sequencing of all isolates from 2012 to 2016. Therefore, for each of these years, a subset of 75% of the received isolates were randomly selected for the study. With this, a total of 356 MRSP isolates were included in the study. Of these, 95% (n = 345) were of canine origin while 5% (n = 11) were from cats. Sample sites represented in the material are shown in Supplementary Table S1.

The samples were inoculated on relevant agar plates (SVA, Uppsala, Sweden) on the day of arrival. Presumptive species identification of isolates based on colony type and morphology, as well as subculture of suspected staphylococcal colonies on bovine blood agar when relevant, was performed prior to the identification of the isolates as *S. pseudintermedius* using matrix-assisted laser desorption ionization time-of-flight mass spectrometry (MALDI-TOF M/S), Bruker Daltonics, Bremen, Germany, as previously described [67,68]. The isolates were confirmed as MRSP by the detection of the *mecA* gene using the qPCR protocol described by [69]. The isolates were stored at −80 °C in trypticase soy broth containing 15% glycerol until further analyses.

### 4.2. Phenotypic Antibiotic Susceptibility Testing

All the MRSP isolates were subjected to susceptibility testing by the determination of minimum inhibitory concentrations (MIC) using broth microdilution, in accordance with recommendations from the Clinical and Laboratory Standards Institute (2013), as previously described by Duse et al. (2021) [70,71]. Colony material was transferred to 5 mL of Mueller-Hinton broth and incubated at 35 °C for three to five hours, after which 3 to 10 µL was transferred into 10 mL of Mueller-Hinton broth, which was subsequently used to inoculate the microtiter plates with 50 µL in each well. The MIC panels were then incubated at 35 °C and read after 16–18 h. Antibiotic substances included and test ranges used are shown in Figure 1. Isolates were classified as either susceptible (wildtype) or resistant (non-wildtype) based on EUCAST epidemiological cut-off values (ECOFFs) when available, otherwise the in-house cut-off values, as presented in published Swedres-Svarm reports [24], were used (Figure 1).

### 4.3. DNA Purification and Sequencing

DNA was isolated from colony material using an EZ1 DNA Tissue Kit (Qiagen, Halden, Germany). Nextera library preparation (Illumina, Foster City, CA, USA) and whole-genome sequencing was performed at Clinical Genomics Stockholm (Science for Life Laboratories, Solna, Sweden) on Illumina Novaseq 6000 (Illumina, Foster City, CA, USA) which produced 2 × 150 bp paired end reads. The raw reads for each sample were quality-checked using FastQC v11.9 [72], trimmed using Trimmomatic v39 [73], and assembled using SPAdes v3.14.0 [74]. The assemblies were error-corrected using Pilon v1.23 [75]. The assembly QC was assessed using SeqSpherePlus v8.3 (Ridom, Würzburg, Germany) (Supplementary Table S2).

### 4.4. Multi-Locus Sequence Typing

A seven-locus multi-locus sequence typing (MLST-7) was performed using the scheme proposed by Solyman et al. [76] available at PubMLST (https://pubmlst.org/organisms/staphylococcus-pseudintermedius, accessed on 7 April 2022), which is based on the seven conserved housekeeping genes *ack*, *cpn60*, *fdh*, *pta*, *purA*, *tuf*, and *sar*.

The assembled contigs for each MRSP isolate were also analyzed using an in-house whole-genome MLST (wgMLST) scheme constructed in SeqSphere+ v8.3 (Ridom, Würzburg, Germany) using the strain E140 [77] as a seed genome and containing 2372 targets from the 2660 genes therein (Supplementary Table S3). The assemblies were questioned against the wgMLST scheme with the following cutoffs: 90% identity and 100% coverage. A minimum spanning tree was constructed from the wgMLST data using Grapetree v1.5.0 [78]. The same wgMLST data was used for calculating a neighbor-joining tree [79] in SeqSphere+ v8.3, visualized in iTol v6 [80].

SCC*mec*Finder v1.2 [81] was used for characterization of SCC*mec* elements. Subtyping of SCC*mec* elements was performed using the Standalone SCC*mec* part of Staphopia [82] and/or manual alignment/mapping of the isolates using BLASTn against the following sequences from GenBank: AB037671.1; AM904732.1; FJ544922.1; AB512767.1; AB505629.1; AB478780.1; AB462393.1; AB121219.1; AB063172.2; AB063173.1; AB096217.1; AB097677.1; DQ106887.1; AB425823.1; AB425824.1; GU122149.1; AB633329.2; AB872254.1; KX385846.1; AB373032.1; MH713898.1; HE984157.2; CP016072.1. The best match was determined by using the highest homology (ID%) and coverage (%).

### 4.5. Identification of Antibiotic Resistance Genes, Stress Response Genes, and Virulence Factors

Antibiotic resistance genes, stress response genes, and virulence factors were identified using AMRFinder+ (https://github.com/ncbi/amr/wiki/Running-AMRFinderPlus, accessed on 2 February 2024) [83] with the *S. pseudintermedius* parameter and the following settings: ≥95% identity and ≥90% coverage. To further search for virulence factors, the assemblies were questioned against the VFDB, accessed on 16 February 2024 [84] and SPVFDB [64] databases using ABRicate (https://github.com/tseemann/abricate, accessed on 4 April 2022) with the following cutoffs: 70% ID and 90% coverage. The sequences were also questioned against the cadmium and zinc resistance gene C, (*czrC*) as described in Aerts et al. 2022 [85] with the following cut-off values: ≥95% identity and ≥90% coverage.

### 4.6. Statistical Analyses

A comparison of the prevalence of the antibiotic resistance to the respective antibiotics between the five most prevalent STs was performed using the chi-square test. *p*-values of <0.05 were considered statistically significant.

## 5. Conclusions

This ten-year-long study of a nationwide collection of MRSP isolates from dogs and cats revealed a significant diversification of MRSP clones present, including both an increasing number of clones and regarding the presence and prevalence of dominant clones succeeding the previously dominant ST71. Several different SCC*mec* elements were found suggesting the independent emergence of various MRSP clones.

All isolates carried an armory of virulence genes encoding factors associated with attachment, colonization, toxin synthesis, quorum sensing, antibiotic resistance, and immune evasion, underlining the risk of a further increase in virulence and antibiotic resistance, and, consequently, a hazard for animals and humans. Antibiotic resistance was highly prevalent to a variety of antibiotic classes, and almost all isolates (96%) were MDR.

## Figures and Tables

**Table 1:** Distributions (%) of MICs for eight antimicrobials against methicillin-resistant *S. pseudintermedius* isolates recovered from dogs and cats in Sweden.

Antibiotic		Non-Wildtype	Distribution (%) of MICs (mg/L)											
	No.	No. (%)	0.12	0.25	0.5	1	2	4	8	16	32	64	128	256
Clindamycin ^a^	360	322 (89.4%)			13.3	1.7	1.4	83.6						
Enrofloxacin ^b^	318	196 (61.6%)		34.0	4.4	1.9	59.7							
Erythromycin ^b^	362	330 (91.2%)			8.8	1.1	90.1							
Fucidic acid ^a^	338	59 (17.5%)			82.5	3.0	3.8	10.7						
Gentamicin ^a^	362	257 (71.5%)				28.5	5.2	13.3	53.0					
Nitrofurantoin ^a^	362	1 (0.3%)								98.1	1.7		0.3	
Sulphonamide–trimethoprim ^a^	362	309 (85.4%)			14.6	5.2	1.1	6.1	72.9					
Tetracycline ^b^	362	244 (67.4%)				32.6	0.3	2.2	64.9					

White fields denote the test ranges for each antibiotic substance. The percentages of isolates with a certain MIC of an antibiotic substance are given in the corresponding field. The percentages shown above the highest test concentration represent isolates with an MIC greater than the highest test concentration. Numbers shown in the lowest test concentration represent isolates with an MIC less than or equal to the lowest test concentration. Vertical bold lines indicate the cut-off values used to define resistance. ^a^ Swedres-Svarm cut-off value. ^b^ EUCAST cut-off value.

**Table 2:** Proportion of non-wildtype (resistant) MRSP isolates within the five most prevalent STs for each of the antibiotic substances included in the study as representatives of respective antibiotic class.

Antibiotic	% Non-Wildtype					
	ST71 (n = 86)	ST258 (n = 42)	ST265 (n = 17)	ST551 (n = 66)	ST45 (n = 7)	Others (n = 138)
Clindamycin	95.3 ^a^	68.3 ^b^	100 ^a^	98.5 ^a^	100 ^a^	78.3 ^a,b^
Enrofloxacin	100 ^a^	0 ^b^	6.7 ^b^	100 ^a^	83.3 ^a c^	40.3 ^c^
Erythromycin	94.2	83.3	100	98.5	100	86.2
Gentamicin	96.5 ^a^	4.8 ^b^	100 ^a^	100 ^a^	100 ^a^	56.5 ^c^
Sulphonamide–trimethoprim	97.7 ^a^	97.7 ^a^	100 ^a^	100 ^a^	71.4 ^b^	68.8 ^b^
Tetracycline	16.3 ^a^	88.1 ^b^	88.2 ^b^	100 ^b^	85.7 ^b c^	73.2 ^c^
Fucidic acid	15.5 ^a^	26.8 ^b^	0 ^c^	4.5 ^c^	0 ^c^	24.8 ^a,b^
Nitrofurantoin	0	0	0	0	0	0.7

Percentages within rows without a common superscript letter: ^a^, ^b^, or ^c^, differ statistically (*p* < 0.05).

**Table 3:** Occurrence of antibiotic resistance genes in 356 *Staphylococcus pseudintermedius* isolates.

Resistance Gene	Antibiotic	No. of Isolates	%
*mecA*	Methicillin	356	100
*blaZ*	Penicillin	343	96.3
*aac(6′)-Ie/aph(2″)-Ia*	Gentamicin, tobramycin	244	68.5
*ant(6)-Ia*	Streptomycin	314	88.2
*ant(9)-Ia*	Spectinomycin	2	0.6
*aph(3′)-IIIa*	Neomycin, kanamycin, paromomycin	317	89.0
*str*	Streptomycin	2	0.6
*spw*	Spectinomycin	38	10.7
*sat4*	Streptothricin	282	79.2
*catA*	Chloramphenicol	83	23.3
*fexA*	Chloramphenicol, florfenicol	3	0.8
*dfrG*	Trimethoprim	289	81.2
*fusC*	Fusidic acid	10	2.8
*erm*(A)	Macrolides, lincosamides	3	0.8
*erm*(B)	Macrolides, lincosamides	317	89.0
*lnu*(A)	Lincosamides	3	0.8
*lnu*(B)	Lincosamides	38	10.7
*lsa*(E)	Pleuromutilins, lincosamides, streptogramin A	38	10.7
*tet*(K)	Tetracyclines	81	22.8
*tet*(L)	Tetracyclines	5	1.4
*tet*(M)	Tetracyclines	216	60.7
*grlA* mutation	Quinolones	222	62.4
*gyrA* mutation	Quinolones	206	57.9

## Data Availability

The data for this study have been deposited in the European Nucleotide Archive (ENA) at EMBL-EBI under accession number PRJEB75029 (https://www.ebi.ac.uk/ena/browser/view/PRJEB75029, publicly available from 9 October 2024.

## Supplementary Materials

The following supporting information can be downloaded at https://www.mdpi.com/article/10.3390/antibiotics13100962/s1, Figure S1: Neighbor-joining tree_antibiotic resistance; Figure S2: Neighbor-joining tree_*SCCmec* and virulence genes; Table S1: Sample sites; Table S2: Sequence QC; Table S3: wgMLST scheme.
