# Supplementary material for: Genomic Analyses of Methicillin-Resistant Staphylococcus pseudintermedius from Companion Animals Reveal Changing Clonal Populations, Multidrug Resistance, and Virulence
Source: Antibiotics (Basel). 2024 Oct 12;13(10):962. doi: 10.3390/antibiotics13100962 (PMC11505346; doi:10.3390/antibiotics13100962)
Supplement: Supplementary file 1 [file Figure S1 Neighbor-joining tree_Resistance genes.pdf › Figure S1 Neighbor-joining tree_Resistance genes.pdf]

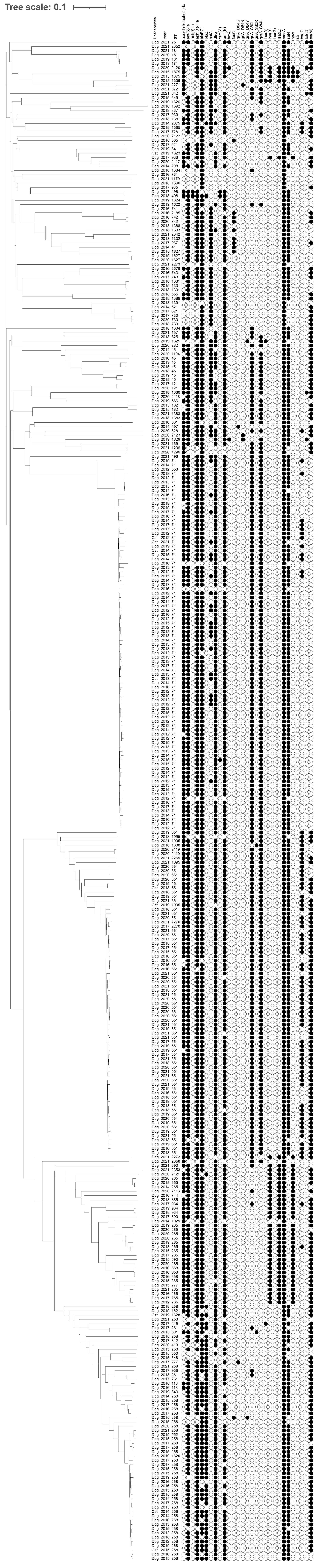
Tree scale: 0.1
Host species
Year
ST
